# Thyroid Cancer Metabolism: A Review

**DOI:** 10.4172/2167-7948.1000200

**Published:** 2016-01-14

**Authors:** Kurren S Gill, Patrick Tassone, James Hamilton, Nikolaus Hjelm, Adam Luginbuhl, David Cognetti, Madalina Tuluc, Ubaldo Martinez-Outschoorn, Jennifer M Johnson, Joseph M Curry

**Affiliations:** 1 Department of Otolaryngology-Head and Neck Surgery, Thomas Jefferson University, Philadelphia, USA; 2 Department of Pathology, Anatomy, and Cell Biology, Thomas Jefferson University, Philadelphia, USA; 3 Department of Medical Oncology, Thomas Jefferson University, Philadelphia, USA

**Keywords:** Papillary, Anaplastic, Thyroid, Cancer, Oxidative phosphorylation, Glycolysis, Mitochondrial metabolism, MCT1, MCT4, TOMM20

## Abstract

Metabolic dysregulation within the tumor microenvironment (TME) is critical to the process of tumorigenesis in various cancer types. Thyrocyte metabolism in papillary and anaplastic thyroid cancer, however, remains poorly characterized, and studies analyzing the role of multicompartment metabolism in thyrocyte oncogenesis are sparse. We present a review of the current knowledge on cellular metabolism in non-cancerous and cancerous thyroid tissues, focusing on the monocarboxylate transporters MCT1 and MCT4, and on a transporter of the outer mitochondrial membrane TOMM20. Understanding the metabolic phenotype of tumor cells and associated stromal cells in thyroid cancer can have profound implications on the use of biomarker staining in detecting subclinical cancer, imaging as it relates to expression of various transport proteins, and therapeutic interventions that manipulate this dysregulated tumor metabolism to halt tumorigenesis and eradicate the cancer. Future studies are required to confirm the prognostic significance of these biomarkers and their correlation with existing staging schemas such as the AGES, AMES, ATA and MACIS scoring systems.

## Introduction

Thyroid cancer is the most common endocrine malignancy in the United States, with the fifth highest incidence of all cancers affecting females [1] and the highest prevalence of any malignancy affecting women under 35 years old. Thyroid cancer has increased in incidence by 5.4-6.5% per year between 2006 and 2010 [1]. Some are predicting it to become the third most common cancer among American women by 2019, surpassing uterine and colorectal cancers [2].

Primary cancer is the most common type of thyroid malignancy, and papillary thyroid cancer (PTC) is the most common histologic type, representing 90% of all thyroid malignancies [2]. Other subtypes include: follicular, medullary, and anaplastic. Prognosis depends greatly on histologic type. Estimated 5-year survival rate for PTC is 98% [2], compared to anaplastic thyroid carcinoma [3], which has a median survival of only 3-5 months [4]. This prognostic disparity emphasizes the importance of classifying the type of thyroid cancer as the primary step in assessment, which is usually diagnosed via fine needle aspiration (FNA). However, cytologic patterns determined by FNA, or even histologic patterns identified by biopsy, may be inconclusive in some cases. Therefore, knowledge regarding the significance of various molecular biomarkers in different metabolic compartments of the tumor can aid in thyroid cancer diagnosis.

Despite a 98% 5-year survival rate, a recent study by Applewhite et al. found that quality of life of thyroid cancer survivors was worse than expected; it was similar to patients with colon cancer, glioma, and gynecologic cancer, and worse than patients with breast cancer [5]. Furthermore, some cases of well-differentiated thyroid cancer are significantly more aggressive than others, making it difficult to predict a patient's course. This heterogeneity of thyroid cancer behavior and unfavorable quality of life for survivors emphasizes the importance of discovering predictive and prognostic biomarkers for thyroid cancer. Once corroborated by future studies, this information can ultimately guide management and impact surgical considerations in patients who are clinically in a gray area of whether to proceed with total thyroidectomy, lobectomy, or, in the case of an indeterminate cancer diagnosis, more conservative measures like close observation and follow-up ultrasounds [6]. In this review, we discuss metabolism in thyroid cancer with an emphasis on our current knowledge of metabolism in the different compartments that constitute the tumor.

### General concepts in tumor metabolism

Dysregulated cellular metabolism has been heralded as one of the “hallmarks of cancer,” which emphasizes its crucial role in oncogenesis [7]. Cancer cells in particular have high bioenergetic requirements needed to promote and maintain cell growth. Carcinoma cells in culture have demonstrated a preferential use of glycolysis with generation of lactate compared to the more energy efficient pathway of oxidative phosphorylation (OXPHOS), which generates more ATP per molecule of glucose than glycolysis [8-12]. This seemingly counterintuitive use of glycolysis may confer a growth and survival advantage, possibly due to increased carbon utilization, hypoxic adaptation, and increased rate of ATP production [8,9,13-16]. This altered metabolism is coined the Warburg effect, which suggests that cancer cells display increased glucose uptake and lactate production irrespective of oxygen availability [17]. This unique glycolytic feature of tumors is the basis of fluoro-2-deoxy-glucose positron emission tomography (FDG-PET) imaging.

**Figure 1:**
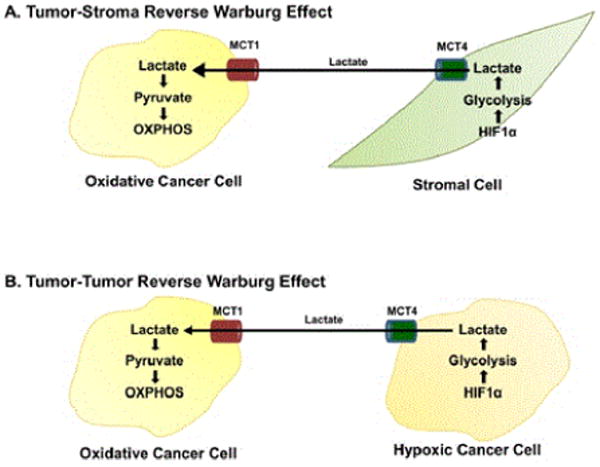
Diagram depicting the reverse Warburg effect in cancer cells and stromal cells (OXPHOS = oxidative phosphorylation, HIF1alpha = hypoxia-inducible factor 1-alpha, MCT1 = monocarboxylate transporter, MCT4 = monocarboxylate transporter 4).

New evidence, however, has shed light on the role of aerobic glycolysis in tumorigenesis, and the Warburg effect has recently become subject to scrutiny, leading to conception of the “reverse Warburg effect.” Aerobic glycolysis occurring in the cancer-associated fibroblasts (CAFs), as opposed to the tumor cells themselves, results in the production of high-energy metabolites such as lactate and pyruvate. Cancer cells at the leading edge of growth exploit nearby glycolytic CAFs to utilize these metabolites, which are ultimately transferred to epithelial cancer cells via inflammation and high levels of reactive oxygen species (ROS) acting as second messengers. Lactate and pyruvate then undergo mitochondrial metabolism, resulting in increased ATP production, which promotes tumor progression. This shift toward aerobic glycolysis occurring in the CAFs and the coupling of different tumoral metabolic compartments is coined the “reverse Warburg effect” [18-22]. This interplay between synergistic metabolic compartments facilitates cancer cell anabolism via the catabolic activity of adjacent tumor fibroblasts (Figure 1) [23].

There has been an increased emphasis on multi-compartment metabolism and metabolic coupling between compartments in the characterization of tumor metabolism. For instance, a two-compartment model of tumor metabolism has been set forth wherein carcinoma cells secrete hydrogen peroxide to induce oxidative stress in tumor fibroblasts or stromal cells [22]. These fibroblasts in turn increase their production of ROS, which induces aerobic glycolysis and autophagy, resulting in increased levels of intermediate catabolites such as lactate, glutamine and ketone bodies [18]. As fibroblasts release these catabolites into the tumor microenvironment, OXPHOS is stimulated in cancer cells [22,24]. Metabolic coupling with glycolysis occurring in some cancer cells and OXPHOS in other cells promotes proliferation and resistance to apoptosis [21].

More recently, a three-compartment model of metabolism has been proposed in normal mucosa and hand and neck squamous cell carcinoma (HNSCC) by Curry et al. In normal mucosa, a hyperproliferative and mitochondrial-rich basal stem cell layer exists that is positive for markers Ki-67, TOMM20, COX, and MCT1. In contrast, the underlying connective tissue and the differentiated epithelial cells are non-proliferative and mitochondrial-poor, making them both negative for MCT4 expression. In HNSCC tumors, the poorly differentiated cancer cells are hyperproliferative (Ki-67+), mitochondrial-rich (TOMM20+/COX+) and use mitochondrial fuels (MCT1+). In contrast, the tumor stroma and well-differentiated cancer cells, the remaining 2 compartments, are non-proliferative and mitochondrial poor. A subset of patients demonstrated MCT4 positivity in these non-proliferating compartments, correlating with a higher disease stage or lower disease free survival [8].

New evidence has identified lactate transporters as being essential to this picture of multi-compartment metabolism. MCT1 is a high-affinity transporter of lactate, which mediates influx into the cell; MCT4 is a low-affinity transporter of lactate that mediates efflux of lactate from cells. These transporters couple cancer cells so that hypoxic cells maintain functioning glycolytic metabolism while aerobic tumor cells recycle and utilize lactate and other high-energy substrates produced by them [13]. It is believed that cells with decreased lactate levels have high lactate uptake via MCTs, allowing them to generate large amounts of ATP via OXPHOS [25,26]. These transporters may represent possible therapeutic targets. MCT4 inhibition by N-acetyl-cysteine, for instance, halts mitochondrial biogenesis in cancer cells but not in normal epithelial cells, ultimately starving cancer cells of required nutrients [27]. MCT1 inhibition prevents uptake of lactate, thereby forcing aerobic cells to use glucose and decreasing availability to hypoxic cells. α-cyanohydroxycinnamate, a MCT1 inhibitor, has been shown to slow tumor growth and potentiate the effect of radiotherapy in MCT1-expressing tumors in mice [13,28].

### General concepts in the tumor microenvironment

Tumorigenesis requires multiple elements as set forth by Hanahan and Weinberg: limitless replicative potential, self-sufficiency in growth signals, insensitivity to anti-growth signals, ability to evade apoptosis, increased angiogenesis, and invasion and metastasis [29]. In order to facilitate these metabolic requirements, solid tumors often reprogram and manipulate their surrounding “condemned tissue,” composed of cancerous cells, adjacent epithelial, stromal, and immune cells and their surrounding matrix. Collectively, these components make up the TME, which serves as a conduit for the cytokine signaling needed to meet the cancer cells' high metabolic requirements. Various signaling pathways, such as NF-kb, HIF-1α, and VEGF continue to be explored as therapeutic targets in the TME [30-32]. In HNSCC, for instance, tumorigenesis has been shown to be driven by signaling pathways such as EGFR, p53, p16, IGFR, cyclin D1, HPV-E6-E7, PI3K-AKT-mTOR, NFkB and HIF-1α [33].

An association between chronic inflammation and increased susceptibility to oncogenesis has been documented for several years, with 20% of all tumors exhibiting persistent low-level inflammation in the TME [34,35]. This has led to the investigation of the roles of various inflammatory mediators and cytokines in cancer cells via intrinsic and extrinsic pathways. The intrinsic pathway begins with activation of oncogenes and/or inactivation of tumor-suppressor genes, and these transformed cells, upon activation by oncogenic signaling pathways, produce inflammatory molecules to generate an inflammatory microenvironment in tumors. On the other hand, in the extrinsic pathway, inflammatory or infectious conditions predispose to cancer development. Both pathways converge to result in activation of transcription factors that coordinate the production of inflammatory mediators that recruit and activate various leukocytes, contributing to the cancer-related inflammatory microenvironment [36].

### Tumor metabolism and the microenvironment in thyroid cancer

Thyroid carcinogenesis exhibits both intrinsic and extrinsic pathway activity. The intrinsic pathway is activated by the most frequent genetic alterations found in PTCs, including RET/PTC, HRAS, or BRAF. These genetic alterations activate, in a RAS-BRAF-MAPK-dependent manner, transcription of proinflammatory molecules like VEGF-A, CXCL1/GRO-α, CXCL10/IP-10, and CXCL8/IL-8, which can act autocrinously or paracrinously to support cancer cell growth and survival [37,38]. The extrinsic pathway involves different immune cells present at the tumor stroma and at the invasive front of thyroid cancer. For instance, high T-regulatory cell density correlates with thyroid cancer aggressiveness, and an increased number of immunoregulatory natural killer cells has been demonstrated in PTC compared to normal tissues [39,40]. Furthermore, is has been shown that the degree of lymphocyte and immature dendritic cell infiltration correlated with better prognosis in PTC [41,42], whereas increased macrophage density in poorly differentiated thyroid carcinoma correlated with invasive features and worsened prognosis [43]. Jung et al. found that tumor-associated macrophage density correlated with more aggressive subtypes, like anaplastic thyroid carcinoma [44]. More recently, a review by Visciano et al. discussed the contribution of mast cells to the epithelial-mesenchymal transformation (EMT) of thyroid cancer cells. They discovered that mast cell-derived mediators like CXCL1/GRO-α and CXCL10/IP-10 are involved in stimulation of cell proliferation, and CXCL8/IL-8 induces/enhances the EMT. Mast cell density was found to correlate with extrathyroidal extension and invasiveness, opening avenues for therapeutic intervention of these mast cell mediators [34]. Lastly, mast cells were significantly more abundant in the intratumoral and peritumoral regions of follicular variant of PTC compared to adenoma, suggesting its usefulness in distinguishing between benign and malignant forms of follicular thyroid lesions [42].

This paradigm of shuttling high-yield metabolites from stromal fibroblasts to fuel cancer cell growth and metastasis has resulted in increased research of various transport proteins. Among these transport proteins are monocarboxylate transporters (MCT), which are a class of membrane bound proteins involved in the influx and efflux of small metabolites such as lactate, pyruvate and ketone bodies [16]. MCT4 is responsible for the export of lactate from CAFs. Lactate is then taken up by cancer cells via MCT1, a bidirectional transporter, and transported to mitochondria via a translocase of the outer mitochondrial membrane (TOMM20), leading to the generation of ATP via OXPHOS [45]. TOMM20 is a central component of the receptor complex responsible for the recognition and translocation of cytosolically-synthesized mitochondrial proteins and has been shown to be an indicator of functional mitochondrial mass and of OXPHOS activity [8,46,47]. Therefore, TOMM20, as well as MCT1, can be used as markers of OXPHOS, and MCT4 a marker of glycolytic metabolism and oxidative stress. Further, these biomarkers have been shown to have prognostic significance: MCT4 is associated with poor outcomes in other cancers [48], and in head and neck cancer specifically, MCT4+ tumor stromal cells were associated with higher tumor stage (p<0.03), poorer clinical outcome (tumor recurrence; p<0.0001) and greater FDG-PET avidity (p<0.04). MCT1 positivity, on the other hand, is prognostic in renal cell cancer and non-small cell lung cancer [49,50] (Table 1).

**Figure 2:**
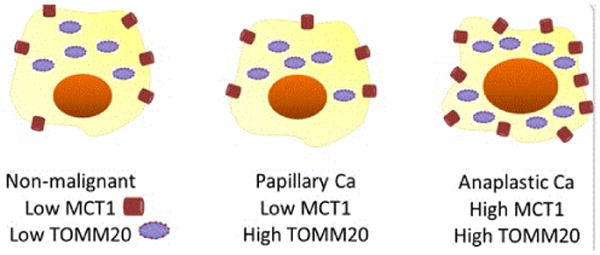
Illustration depicting low MCT1 and TOMM20 in non-malignant thyroid tissue, low MCT1 and high TOMM20 in PTC, and high MCT1 and TOMM20 in ATC.

Our group previously characterized tumor metabolism in thyroid cancers specifically looking at TOMM20, MCT4 and MCT1. We interrogated, by IHC, non-cancerous, PTC, and ATC tissue for these biomarkers, and review our discoveries on the metabolic profiles of these tissue types. In all non-tumor thyroid tissue and multinodular goiter samples, TOMM20 expression was low. Fibroblasts in NCT and NG specimens demonstrated low MCT4 expression as well [51] (Figure 2). No NCT samples had high expression of MCT1 (p<0.0001) [3]. In summary, IHC of NCT and NG tissue samples demonstrated low staining of all 3 biomarkers: stromal MCT4, cancer cell TOMM20 and cancer cell MCT1. In follicular adenoma specimens, all of the adenomatous thyrocytes demonstrated high expression of TOMM20 compared to adjacent non-tumor thyrocytes and nodular goiter samples [51]. The fibroblasts around the adenoma and throughout the rest of the gland showed low MCT4 staining. In one case, MCT4 was elevated around the adenoma, but negative throughout the rest of the gland [51].

### Papillary Thyroid Cancer (PTC)

All PTC thyrocytes from patients with and without advanced disease showed homogeneously high expression of TOMM20 throughout the tumor. Of note, there was a difference between intensity of staining between non-advanced and advanced PTC specimens, but this was not statistically significant (p=0.36) [52]. Specimens from the PTC group with advanced disease demonstrated higher MCT4 staining in the CAFs compared to PTC without advanced disease group; this difference was statistically significant (p<0.01). MCT1 expression was low in PTC specimens (p<0.001).

### Anaplastic Thyroid Cancer (ATC)

There was significantly more TOMM20 staining in ATC compared to NCT (p<0.05) [3], and majority of samples also showed robust MCT1 expression.

## Discussion

### Current limitations in thyroid cancer diagnosis - Unmet clinical needs

Palpable thyroid nodules occur in 4-7% of the population [53-58], and 19-67% of lesions are found incidentally during ultrasonographic examination [6]. Management of thyroid nodules poses a significant challenge. Tools such as FNA cytology have helped considerably in differentiating benign from malignant thyroid lesions. However, this is not without limitations as it can be quite difficult to assess malignancy on cytology, which has necessitated the Bethesda criteria for risk stratification of thyroid aspirates [59]. Secondary to the shortcomings of thyroid FNA, many patients undergo diagnostic surgery, after which less than 30% of lesions are diagnosed as malignant [60]. These inadequacies of FNA have placed an emphasis on proteomic and genomic research in thyroid cancer. The BRAF gene mutation, which has been detected in 30-80% of PTC [61], has been an area of interest to improve the diagnostic yield in cases of indeterminate cytology. The BRAFV600E mutation is very specific for PTC; however, its absence cannot reliably rule out PTC [3].

### Summary

Histopathological evaluation remains the gold standard in distinguishing different types of thyroid cancer, but is also subject to inaccuracies. For example, several morphologic features, such as nuclear atypia or pleomorphism, can be seen in Hashimoto's thyroiditis, and are not sufficient for a reliable diagnosis of malignancy [6]. Advances in IHC have attempted to enhance histopathologic diagnostic accuracy. One challenge of proteomic studies of thyroid tissue, however, is that thyroid tissue is extremely heterogeneous, with a broad range between the most abundant proteins, such as thyroglobulin, and the least abundant proteins [6]. In differentiating between follicular adenoma, follicular carcinoma, and the follicular variant of PTC, several biomarkers have proved to be applicable, including LGALS, hemoglobin, epsilon 1 (HBE1), cytokeratin-19 (CK-19), and thyroid peroxidase (TPO) [62]. Yet, these too are not without their diagnostic inaccuracies, as 31-55% of adenomatous hyperplasia could be positive for CK-19. Expression of other proteins, such as S100A, peroxiredoxin, and heat shock protein 70 (HSP70), were shown to be significantly increased in PTC specimens matched with the normal thyroid tissue from the same patients [63]. Poorly differentiated thyroid carcinoma (PDTC) exhibits diffuse nuclear positivity for thyroid transcription factor 1 (TTF1) and focal positivity for thyroglobulin. On the other hand, undifferentiated thyroid carcinomas, such as anaplastic carcinoma, are by definition thyroglobulin negative and almost always TTF1 negative [64].

In spite of the advances made in proteomic characterization of thyroid cancer cells, information about how this correlates to prognosis remains indeterminate. This introduces different avenues to identify other biomarkers with greater prognostic yield. Among these biomarkers are monocarboxylate transporters MCT1 and MCT4, and a translocase of the outer mitochondrial membrane TOMM20. These and other markers may offer some diagnostic benefit in the future.

### Clinical applications of thyroid cancer metabolism and future research directions

The preliminary knowledge of thyroid cancer metabolism discussed in this review may serve as a stepping-stone to advancements in thyroid cancer diagnosis and management. With further studies corroborating and expanding on our findings, biomarkers such as TOMM20, MCT4 and MCT1 can serve as early prognostic indicators in thyroid tissue samples of patients suspicious for cancer. For instance, no tumors with low fibroblast MCT4 staining demonstrated advanced disease or aggressive features. Furthermore, low fibroblast MCT4 staining was associated with lower MACIS, AMES, AGES and ATA risk levels and the absence of extrathyroidal extension [51], which enhances the prognostic significance of these biomarkers.

These data may also have implications on imaging of thyroid cancers. A study by Curry et al. demonstrated that MCT4+ staining was specific for tumor tissue and oxidative stress leading to carcinogenesis, which in turn correlated with high PET avidity and poor clinical outcomes [8]. PET scans, initially thought to be detecting glucose uptake in cancer cells, are now being re-interpreted as detecting the “reverse Warburg effect” in CAFs. CAFs show the largest increase in glucose uptake, while cancer cells exhibit corresponding decreases in glucose uptake under identical co-culture conditions. These novel findings suggest that PET imaging detects the tumor stroma rather than the epithelial cancer cells [22]. The relationship between tumor glycolysis as measured by MCT4 staining and tumor uptake on PET scans specifically for thyroid cancer has not previously been studied and is a potential area for future research.

These findings may also impact surgical considerations in thyroid carcinoma. The mainstay treatment of PTC is surgical resection, but controversy persists over whether total thyroidectomy or thyroid lobectomy is more beneficial [2]. A review of 31 patients with PTC undergoing conservative surgery of lobectomy with isthmusectomy identified a rate of 20% contralateral recurrence after a 20-year follow-up, and therefore supports total thyroidectomy as the initial therapy [65]. Assessing thyroid cancer aggressiveness by identifying the tumor cell metabolic phenotype can also have implications on the cost of care of thyroid cancer patients in the United States. Currently, the costs of management of PTC are substantially greater in the US compared with other countries. One study comparing 100 US and 100 French PTC patients showed that the median cost per patients was greater in the US than in France ($1,069 *vs*. $,590, p<0.001) despite the length of stay being shorter in the US (1 *vs*. 3 days, p<0.001). Total thyroidectomy with central neck dissection is also more frequently done in the US (92% *vs*. 35%, p<0.001). IHC staining of prognostic biomarkers in tissue samples of patients with suspected thyroid cancer may lead to less aggressive and expensive intervention later on by detecting these patients earlier on and managing them more conservatively [66].

Targeting the metabolism of cancer cells or their adjacent i with mitochondrial inhibitors such as metformin may offer a novel therapeutic strategy for PTC and ATC. Metformin is a mitochondrial complex I inhibitor that blocks mitochondrial-dependent ROS production and leads to decreased ATP production. Extensive preclinical data supports the efficacy of metformin as an antineoplastic agent [67], inhibiting cancer cell proliferation in gastric, medullary thyroid, breast, and pancreatic cancers [68-71]. Epidemiologic studies have also shown a decreased risk of cancer incidence and mortality from metformin use in diabetics [72], and clinical trials elucidating metformin's effect on head and neck squamous cell carcinoma are already underway. Further studies assessing its role in thyroid cancer, among other cancers, are needed to support these claims. A future area of interest would also be the development of other MCT1 and MCT4 inhibitors in order to limit tumoral ATP production [73].

## Conclusions

We present a review of the current knowledge of metabolism in thyroid cancer, integrating our recent discoveries on the role of transmembrane lactate transporters MCT1 and MCT4, and a translocase of the outer mitochondrial membrane TOMM20. PTC samples exhibited TOMM20 and MCT4 positivity, whereas the more aggressive ATC demonstrated robust TOMM20 and MCT1 positivity. This contrasts non-cancerous and nodular goiter thyroid tissue, which were negative for all three biomarkers. Our characterization of the multiple metabolic compartments in thyroid cancer subtypes opens up avenues for therapy by intervening in the pathway to ATP generation with mitochondrial inhibitors like metformin. These novel findings also have prognostic significance that can help guide management in certain patients. Further studies are needed to corroborate these results and bolster the significance of these claims.

## Figures and Tables

**Table 1:** Table outlining the immunohistochemical staining patterns of TOMM20, MCT1 and MCT4 in noncancerous and cancerous thyroid tissue specimens.

	NCT/NG	FA	PTC	ATC
TOMM20 (in thyrocytes)	–	+	+	+
MCT4 (in CAFs/stroma)	–	–	+	
MCT1 (in thyrocytes)	–		–	+

(TOMM20 = transporter of the outer mitochondrial membrane 20, NCT/NG = noncancerous thyroid/nodular goiter, FA = follicular adenoma, PTC = papillary thyroid cancer, ATC = anaplastic thyroid cancer).
